# Supplementary material for: Reduced Replication of Highly Pathogenic Avian Influenza Virus in Duck Endothelial Cells Compared to Chicken Endothelial Cells Is Associated with Stronger Antiviral Responses
Source: Viruses. 2022 Jan 17;14(1):165. doi: 10.3390/v14010165 (PMC8779112; doi:10.3390/v14010165)
Supplement: Supplementary file 1 [file viruses-14-00165-s001.zip › viruses-1523307-supplementary.pdf]

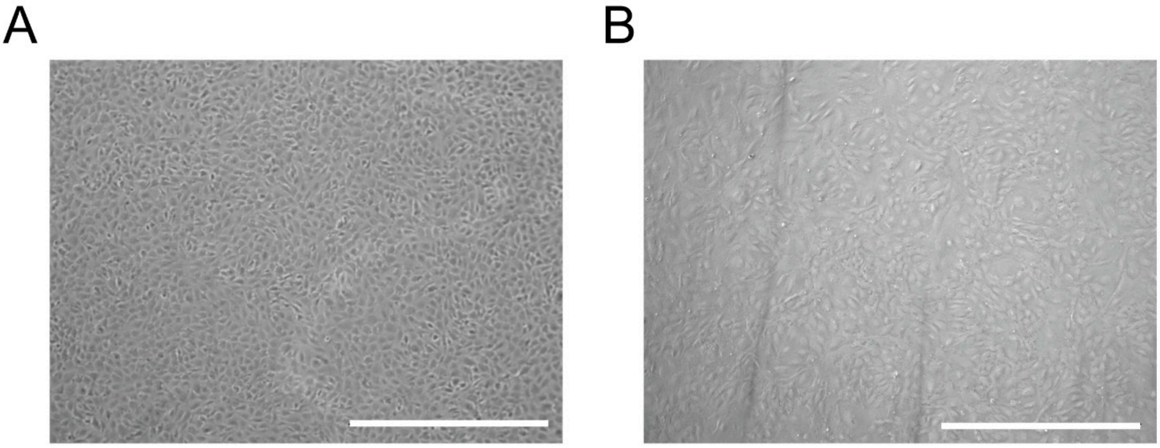

Supplementary Figure S1. Primary AEC have an endothelial cell morphology. Bright field microscopy pictures of chAEC p14 (A) and dAEC p15 (B). The scale bar represents 1 mm.

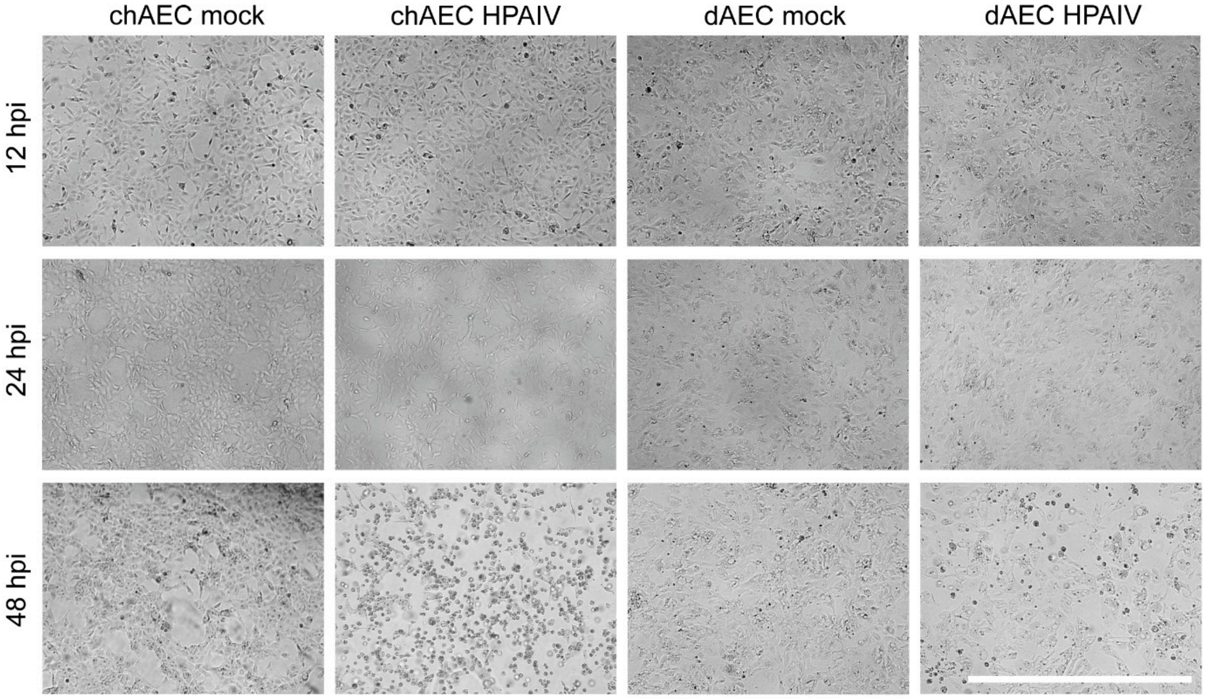

Supplementary Figure S2. Delayed onset of cytopathic effects in dAEC compared to chAEC upon inoculation with HPAIV. Representative bright field microscopy images of the multi-cycle replication experiment in Figure 3 panel C (see figure legend for details). The scale bar represents 1 mm.
